# A Design Method to Induce Ductile Failure of Flexural Strengthened One-Way Reinforced Concrete Slabs

**DOI:** 10.3390/ma14247647

**Published:** 2021-12-12

**Authors:** Huy Q. Nguyen, Tri N. M. Nguyen, Do Hyung Lee, Jung J. Kim

**Affiliations:** 1 Department of Civil Engineering, Kyungnam University, Changwon-si 51767, Korea; nguyenquochuy@muce.edu.vn; 2 Campus in Ho Chi Minh City, University of Transport and Communications, No. 450-451 Le Van Viet Street, Tang Nhon Phu A Ward, Thu Duc City, Ho Chi Minh City 700000, Vietnam; trinnm_ph@utc.edu.vn; 3 Department of Civil, Railroad and Unmanned Systems Engineering, PaiChai University, 155-40 Baejaero, Seo-gu, Daejeon 35345, Korea; dohlee@pcu.ac.kr

**Keywords:** reinforced concrete slab, end span, flexural failure, failure modes, design methodology, continuous slab

## Abstract

Strengthening existing reinforced concrete (RC) slabs using externally bonded materials is increasingly popular due to its adaptability and versatility. Nevertheless, ductility reduction of the rehabilitated flexural members with these materials can lead to brittle shear failure. Therefore, a new approach for strengthening is necessary. This paper presents a methodology to induce ductile failure of flexural strengthened one-way RC slabs. Ultimate failure loads can be considered to develop the proposed design methodology. Different failure modes corresponding to ultimate failure loads for RC slabs are addressed. Flexural and shear failure regions of RC slabs can be established by considering the failure modes. The end span of the concrete slab is shown for a case study, and numerical examples are solved to prove the essentiality of this methodology.

## 1. Introduction

The demand for strengthening and rehabilitation of infrastructures becomes more urgent in modern society [1,2,3]. Apart from the aging, corrosion, unexpected excessive loads, and accidental damage, the rectify of initial design and construction faults or upgrading the load capacity of reinforced concrete (RC) structures are also of interest to structural engineers and researchers [4,5,6]. Moreover, strengthening the existing structures is preferable to demolishing and building an entirely new system due to its lower costs and minimized environmental deterioration simultaneously [7].

**Figure 1:**
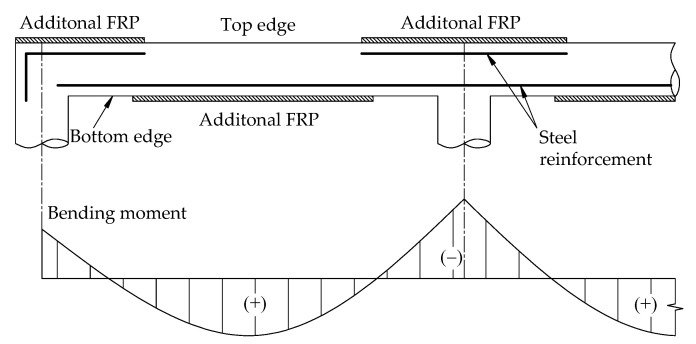
Externally bonded FRP strengthened slab at conventional locations.

Repairing or strengthening RC structures using reinforced concrete, ferrocement, steel, or fiber reinforced polymer (FRP) materials on the tension parts of the RC structures is one of the most common techniques [8]. In addition, the development of FRP composite materials allows improving significant loading capacity of strengthened structures [9,10,11,12,13]. Over four decades, several studies have been solved to examine the performance of the structures strengthened with FRP and proven its effectiveness due to its high tensile strength and corrosion resistance [14,15,16,17,18]. The post strengthening with FRP materials can rehabilitate the original flexural capacity of the damaged structures and even increase it significantly. There are many methods for post strengthening flexural RC slabs with FRP [19,20,21,22]. One of the typical methods is the addition of FRP on the top and bottom parts of the slabs subjected to the positive (+) and negative (–) flexural moments, respectively, as shown in Figure 1. However, the brittle failure and the ductility reduction of the rehabilitated flexural members were recognized [23,24]. 

Additionally, RC structures in contact with FRP composite materials have significant and unavoidable effects on their behavior [25]. The failure mode of strengthened RC structures tends to be more brittle compared with the counterpart steel-reinforced concrete structures due to the intrinsic bond conditions between FRP and concrete and the linear-elastic brittle tensile behavior of FRP as well [26]. Structural ductility should be considered an important design factor because it can prevent brittle shear modes [27,28]. A well-designed structure should warn of impending failure when it is subjected to an overload [29]. Several studies have shown the necessity of failure modes in reflecting the corresponding behavior of the structure under load [30,31,32]. Although some studies have been investigated on the failure modes of strengthened structures [33,34,35,36], there is little reported work on strengthened flexural structures avoiding sudden failure and inducing ductile failure. 

This research work recommends using carbon fiber reinforced polymer (CFRP) unidirectional laminates to enhance the strength of existing continuous slabs. However, the excessive improvement of the flexural strength relative to the shear strength of the strengthened sections can lead to brittle shear failure [37]. To the best of our knowledge, there is not yet a complete design process of preventing brittle failure for strengthened RC slabs. This study proposes a new classification of failure modes that reflect the corresponding behavior of the RC slab under load. Structural evaluation through new failure modes can suggest the appropriate enhancement of the existing RC slabs. Based on that, the methodology limiting the additional strength for post-strengthened RC slabs through the failure modes to prevent brittle failure and induced ductile failure is also determined.

In this paper, different failure modes for the end span of a continuous RC slab corresponding to ultimate failure loads for RC slabs are addressed for a case study. The results would contribute to developing a design methodology for the strengthened RC slab to ensure ductile failure.

## 2. Failure Limits

**Figure 2:**
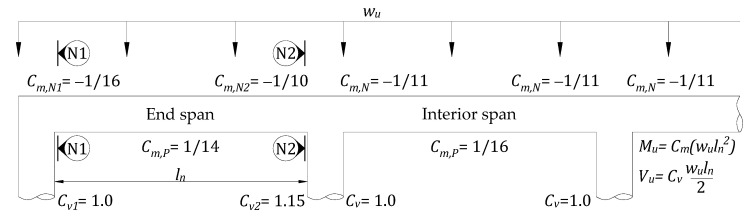
Moment and shear coefficients for continuous RC slabs with column supports reported by ACI 318M.

This work focuses on determining the failure limits of the end span of a continuous slab subjected to uniformly distributed load. For flexural members in a frame, the flexural rigidities of members and supporting columns are decisive factors in the distribution of bending moments. Thus, the moment at the support and the mid-span sections of the frame members, subjected to a uniformly distributed load w, could be established, as $M=Cwl^{2},$ where *C* is a coefficient according to flexural rigidities of respective flexural members and *l* is the clear span length. If infinite rigidity of columns is considered, the well-known results for a fixed-end moment of a flexural member subjected to a uniformly distributed load w, coefficient *C* will be 1/12 [38]. For practical design purposes, moment and shear coefficients for continuous RC slabs subjected to a uniformly distributed load ($w_{u}$) are reported by ACI 318M [39] with column support cases, as shown in Figure 2.

Considering a flexural member having unsymmetric boundary conditions, which is comparable to the end span in Figure 2, the different failure modes could be established according to the relationship between the moment limit of the negative moment *M_n,N_* and positive moment *M_n,P_* at the support and the mid-span sections, respectively, and the shear limit of *V_n_* of the slab sections. In case the sections at the mid-span and the exterior face of the first interior support (N2) fail simultaneously by forming two plastic hinges, the moment limits subjected to a uniform distributed load can be written as a formula of the shear carrying capacity, *V_n_*, as follows:


(1) $$M_{n,N}=\frac{2C_{m,N2}}{C_{v2}}V_{n}l_{n}$$

(2) $$M_{n,P}=\frac{2C_{m,P}}{C_{v2}}V_{n}l_{n}$$

At the same time, the moment carrying of the interior face of the exterior support (N1) can be expressed as follows,

(3) $$M_{N1}=\frac{2C_{m,N1}}{C_{v2}}V_{n}l_{n}$$

where *M_n,N_* and *M_n,P_* are the moment carrying capacity of the support and mid-span sections; *M_N1_* is the moment carrying of the N1 section; *V_n_* is the shear carrying capacity of the RC slab sections; *C_m,N_*_1_, *C_m,N_*_2_, and *C_m,P_* are the moment coefficients for the negative moments at the N1 section, N2 section, and the positive moment at the mid-span section, respectively; *C_v2_* is the shear coefficient at the N2 section; and *l_n_* is the clear span length between support columns. 

**Figure 3:**
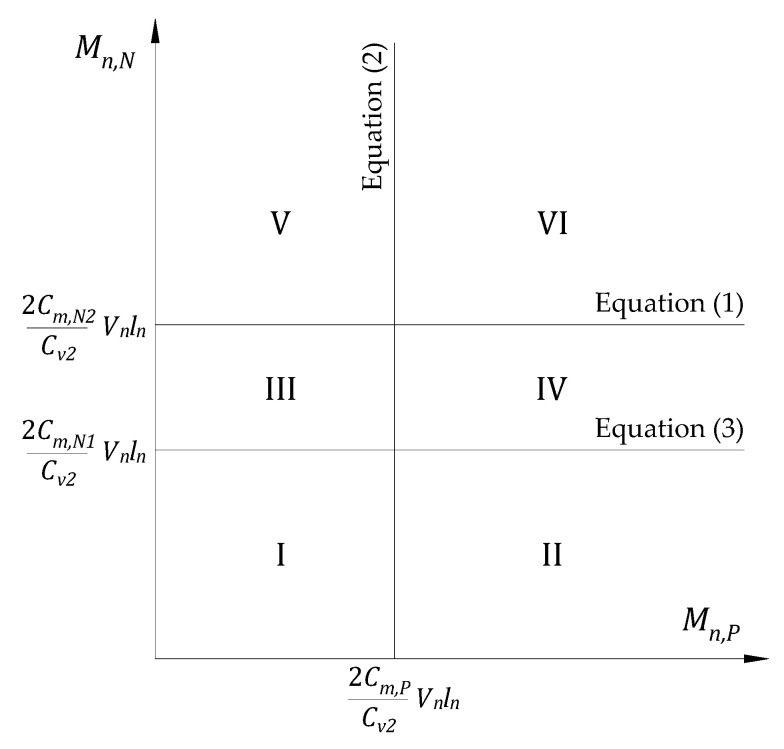
The failure regions are described considering the relationship between the shear limit and moment limit of the slab sections.

Here, the signs of the moment coefficients could be neglected because they are only used to show moment directions, as shown in Figure 2. The limit of Equations (1)–(3) may be described with a given shear limit and the shear and moment coefficients (refer to Figure 3). The failure regions of RC slabs could be shown as follows.

Failure modes should be classified according to the order of plastic hinge formation at sections and the type of failure by analyzing the failure limit for each region in Figure 3. The summary of different failure modes is shown in Table 1. Details of the analysis for Table 1 are described in the Appendix A of this article. Failure modes of D-1, D-2, and D-3 are ductile failures and desirable while the failure modes DB-1, DB-2, DB-3a, DB-3b, B-1, and B-2 are brittle failures and thus may not be suitable for a well-designed structure. 

**Figure 4:**
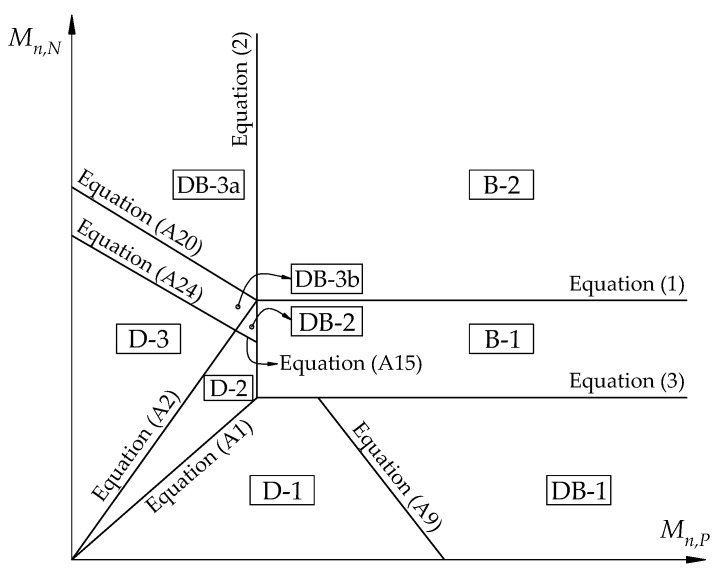
Different failure modes according to *M_n,P_, M_n,N_,* and *V_n_* for the end span of an RC slab.

The limit equations of failure modes in each region are shown in Figure 4. The ultimate failure load can be determined by the superposition method considering plastic redistribution of strengthened RC slab. For failure modes of D-1, D-2, and D-3, the ultimate failure loads can be calculated as:

- Failure mode D-1

(4) $$w_{f}=\phi_{m}\frac{8}{l_{n}^{2}}\left(M_{n,P}+M_{n,N}\frac{\left(1/8-C_{m,P}\right)}{C_{m,N2}}\right)$$

- Failure mode D-2

(5) $$w_{f}=\phi_{m}\frac{4}{l_{n}^{2}}\left(M_{n,P}+M_{n,N}\frac{\left(1/4+C_{m,N2}-C_{m,N1}-C_{m,P}\right)}{C_{m,N2}}\right)$$

- Failure mode D-3

(6) $$w_{f}=\phi_{m}\frac{4}{l_{n}^{2}}\left(M_{n,P}\frac{\left(1/4-C_{m,N1}\right)}{C_{m,P}}+M_{n,N}\right)$$

For failure modes of DB-1, DB-2, DB-3a, DB-3b, B-1, and B-2, the failure loads can be calculated as:

(7) $$w_{f}=\phi_{v}\frac{2V_{n}}{C_{v2}l_{n}}$$

where strength reduction factors of $\Phi_{m}\;\mathrm{and}\;\Phi_{v}$ are used for the flexural strength and the shear strength, respectively, as specified by ACI 318M [39].

## 3. Design Example 

**Figure 5:**
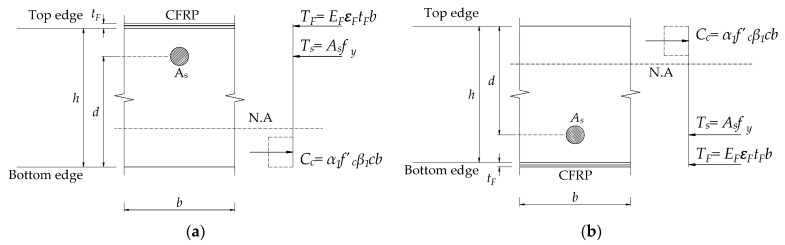
The CFRP strengthened sections are subjected to (**a**) negative moment and (**b**) positive moment.

**Figure 6:**
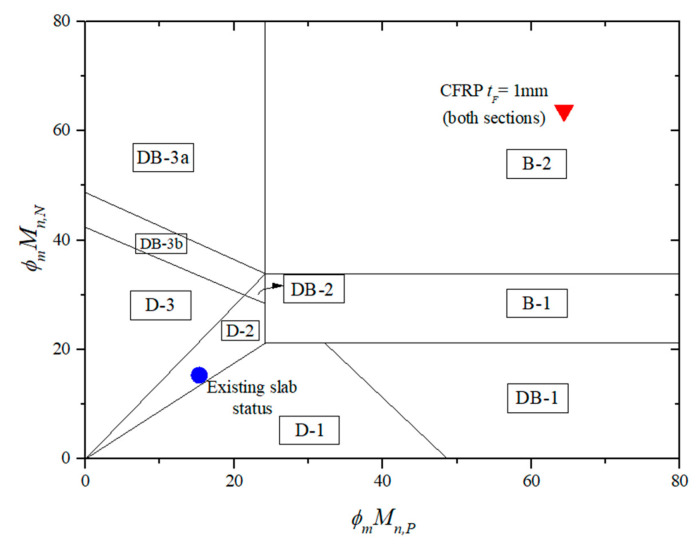
Failure limits for the flexural strengthened slab with CFRP.

In this case, an RC slab strengthened with an externally bonded CFRP sheet has been presented. Moment and shear coefficients are considered for the end span of the column support case, where $C_{m,N1}=1/16,\;C_{m,N2}=1/10,\;C_{m,P}=1/14,\;C_{v1}=1,\;$ and $C_{v2}=1.15,$ as shown in Figure 2. The strength reduction factors of the flexural strength and shear strength are 0.90 and 0.75, respectively [39]. The reduction factor for the strength contribution of CFRP reinforcement *ψ_f_* is 0.85 [40]. The selected CFRP along with mechanical properties (tensile strength *f_fu_* = 717 MPa, elastic modulus E_f_ = 65.1 GPa) is reported by the manufacturers, as recommended by ACI 440R [41]. Additional CFRP thickness (*t_F_*) is assumed as a design variable and installed in tensile regions of the RC slab corresponding to the width of the slab (refer to Figure 5). The clear span of the rectangular RC slab is 2.5 m long. Slab material properties and dimensions are summarized in Table 2. The existing slab is computed in Table 3, and the failure mode considering the relationship between factor flexural and shear resistance and slab status is extracted, as shown in Figure 6. In this analysis, a perfect bond between the strengthened materials and the RC slab is supposed up to the ultimate failure loads. 

## 4. Results and Discussions

For the calculation of the existing slab shown in Table 3, the ultimate failure load ($w_{f}$) of the existing slab is determined as 31 kN/m, as indicated by Equation (5). The failure mode of the slab is named D-2, as shown in Figure 6. Notably, the existing slab can continue to be strengthened to also fail in ductile failure. The calculation procedure for 1 mm thick CFRP sheet strengthened for positive and negative moment sections of the RC slab is shown in Table 4, as specified by ACI 440 [40]. In step 2, the existing state of strain is determined. CFRP debonding is computed at step 3 and is used in steps 4 to 8 and shows that concrete strain is less than the failure strain of 0.003. In this case, debonding of CFRP occurs before the failure strain of concrete reaches *ε* = 0.0021. The coefficients of rectangular stress block *α_1_* and *β_1_* for the failure strain in the concrete at the limit state are applied. The design flexural and shear strength are calculated in steps 9 through 12, whereas the ultimate failure load ($w_{f}$) is computed in steps 13 through 14 with corresponding failure mode. The failure mode of the slab is named B-2, as shown in Figure 6, with the ultimate failure load $w_{f}$ = 54.2 kN/m using Equation (7). Although the ultimate failure load is increased 75% compared to the ultimate failure load of the existing slab, brittle failure is not a desirable result. 

**Figure 7:**
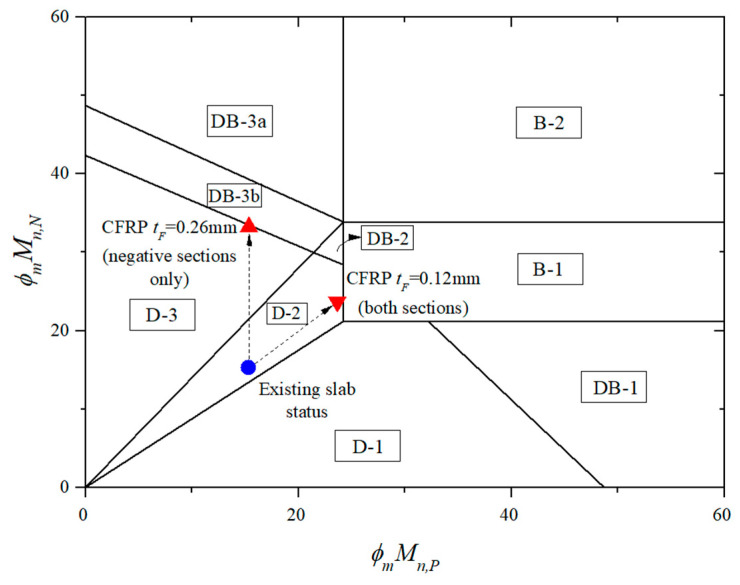
Failure limits for the flexural strengthened slab with CFRP considering ductile failure.

A similar calculation procedure is performed while the CFRP sheet thickness is assumed as a design variable and adjusted to ensure ductile flexural failure. The existing slab can be reinforced by installing 0.12 mm thick CFRP sheets for positive and negative moment sections. The ultimate failure load of the strengthened slab $w_{f}$, found from Equation (5), is estimated as 47.9 kN/m, which increased by 55% compared to the ultimate failure load of the existing slab. The failure mode is named D-2, as shown in Figure 7.

In another approach for ease of application [19], a 0.26 mm thick CFRP sheet could be applied at the upper side of the slab to enhance negative moment capacity. The failure mode is named D-3, as shown in Figure 7. Using Equation (6), the ultimate failure load of the strengthened slab $w_{f}$ is determined as 47.1 kN/m, which is increased by 52% compared to the ultimate failure load of the existing slab. 

The flexural strengthened RC slab capacities are shown in Table 5. From this table, one can realize that the strengthened slab with 0.12 mm thick CFRP for positive and negative sections and 0.26 mm thick CFRP for only negative parts has the efficiency to enhance the factored design load by 155% and 152%, respectively. Especially, the failure mode of the strengthened RC slab is a desirable ductile failure to which there is little reported concern to prevent sudden failure.

## 5. Conclusions

Failure modes of the continuous RC slab considering the relationship between the flexural strength and shear strength to prevent brittle shear failure and induce flexural ductile failure are presented. 

An efficient procedure to strengthen slabs flexurally with an externally bonded CFRP avoiding sudden failure and ensuring ductile failure is demonstrated through several examples. By using 0.12 mm thick CFRP for positive and negative sections and 0.26 mm thick CFRP for only negative parts of the slab, the factored design load can be enhanced by 155% and 152%, respectively.

A simple approach to determine the additional strength limit for flexural slabs considering failure modes is introduced. Adjusting the thickness of the CFRP sheet could achieve the desired increase in flexural strength. Additionally, CFRP discrete strips may also be used instead of CFRP sheets if their thickness is fixed by manufacturers.

The method has advantages in constructability and economic aspects. It can be applied to strengthen the existing floor slabs or bridge decks. Furthermore, the additional flexural strength with an externally bonded CFRP could be also optimized with a warning of impending failure under overload. 

This study is theoretical, and it would contribute to developing a design methodology for the strengthened RC slab to ensure ductile failure, completed through finite element analysis and experimental research in further studies.

## Figures and Tables

**Table 1:** Summary of different failure modes for the end span of an RC slab.

Failure Modes	First Plastic Hinge	Second Plastic Hinge	Third Plastic Hinge	Shear Failure	Failure Type
D-1	N2	N1	M	-	Ductile
D-2	N2	M	N1	-	Ductile
D-3	M	N2	N1	-	Ductile
DB-1	N2	N1	-	N2	Brittle
DB-2	N2	M	-	N2	Brittle
DB-3a	M	-	-	N2	Brittle
DB-3b	M	N2	-	N2	Brittle
B-1	N2	-	-	N2	Brittle
B-2	-	-	-	N2	Brittle

**Table 2:** The RC slab material properties and dimensions for design example.

Section	*h* (mm)	*b* (mm)	*A_s_* (mm^2^)	*d* (mm)	*f’_c_* (MPa)	*γ_c_* (kg/m^3^)	*f_y_* (MPa)	*E_s_* (GPa)
Supports	150	1000	355	120	27	2400	410	200
Mid-span								

**Table 3:** Calculation of the existing RC slab.

Calculation	Existing RC Slab
Moment and shear coefficients for the end span of the column support case [39]	$C_{m,N1}=1/16,\;C_{m,N2}=1/10,\;C_{m,P}=1/14\;C_{v1}=1,\;\mathrm{and}\;C_{v2}=1.15$
Designed resistance	$\phi_{f}M_{n,P}=15.3\;\mathrm{kNm},\;\phi_{f}M_{n,N}=15.3\;\mathrm{kNm},$ $\phi_{v}V_{n}$ = 77.94 kNm;
Failure mode	D-2, as shown in Figure 6
Ultimate failure load: Equation (5) for D-2	$\begin{array}{l} w_{f}=\frac{4}{2.5^{2}}\left(15.3+15.3\frac{\left(1/4+1/10-1/16-1/14\right)}{1/10}\right)\\ \;\;=31\;\mathrm{kN}/m \end{array}$
Preparatory computations for strengthened design	
Self-weight $w_{D}=\gamma_{c}bh$	$w_{D}=(2400)(9.8\times 10^{-3})(0.15)=3.53\;N/\mathrm{mm}$
Factored moment $M_{D}=C_{m}w_{D}l_{n}^{2}$	At N2 section $M_{D,N}=(1/10)(3.53)(2500^{2})/1000=2205\;\mathrm{kNmm}$ At mid-span section $M_{D,P}=(1/14)(3.53)(2500^{2})/1000=1575\;\mathrm{kNmm}$
Modulus of elasticity $E_{c}=4700\sqrt{f_{c}^{\prime }}$	$E_{c}=4700\sqrt{27}=24400\;\mathrm{MPa}$
Cracking moment $I_{cr}$ Neutral axis depth *c* = *kd*	At both sections (N2 and mid-span sections) $I_{cr}=31.4\times 10^{6}{\;\mathrm{mm}}^{4}$ c=7.5 mm

**Table 4:** Calculation of the strengthened slab.

Procedure	Strengthened Slab
1. CFRP thickness, *t_F_*	At both sections: *t_F,N_ = t_F,P_* = 1 mm
2. Existing state of strain $\varepsilon_{bi}=\frac{M_{D}\left(d_{f}-kd\right)}{I_{cr}E_{c}}$ *kd* is *c* in Table 3	At N2 section: $\varepsilon_{bi,N}=\frac{(2.205\times 10^{6})\left(150.6-7.5\right)}{\left(31.4\times 10^{6})(24400\right)}=0.00041$ At mid-span section: $\varepsilon_{bi,P}=\frac{\left(1.575\times 10^{6})(150.6-7.5\right)}{\left(31.4\times 10^{6})(24400\right)}=0.00029$
3. Design strain of CFRP $\varepsilon_{fd}=0.41\sqrt{\frac{f_{c}^{\prime }}{nE_{F}t_{F}}}\leq 0.9\varepsilon_{fu}$	$\varepsilon_{fd}=0.41\sqrt{\frac{27}{\left(1)(65100)(1\right)}}=0.0083<0.9(0.011)=0.0099$
4. Assume concrete strain at failure *ε* Revise *ε* until equilibrium achieved	At both sections: $\varepsilon_{c,N}=\varepsilon_{c,P}=0.0021$
5. Compute neutral axis depth	At both sections: $c_{N}=c_{P}=32.7\;\mathrm{mm}$
6. Compute CFRP strain $\varepsilon_{fe}=\varepsilon_{c}\left(\frac{h-c}{c}\right)-\varepsilon_{bi}\leq \varepsilon_{fd}$	At N2 section: $\varepsilon_{fe,N}=0.0021\left(\frac{150-32.7}{32.7}\right)-0.00041=0.0072<0.0099$ At mid-span section: $\varepsilon_{fe,N}=0.0021\left(\frac{150-32.7}{32.7}\right)-0.00029=0.0073<0.0099$
7. Compute tension steel strain $\varepsilon_{s}=\varepsilon_{c}\left(\frac{d-c}{c}\right)$	At both sections: $\varepsilon_{s,N}=\varepsilon_{s,P}=0.0021\left(\frac{120-32.7}{32.7}\right)=0.0056>0.002\;\mathrm{yield}$
8. Check for force equilibrium $\beta_{1}=\frac{4\varepsilon_{c}^{\prime }-\varepsilon_{c}}{6\varepsilon_{c}^{\prime }-2\varepsilon_{c}}$, $\alpha_{1}=\frac{3\varepsilon_{c}^{\prime }\varepsilon_{c}-\varepsilon_{c}^{2}}{3\beta_{1}{\varepsilon^{\prime }}_{2}^{c}}$ $\varepsilon_{c}^{\prime }$ is strain relative to $f_{c}^{\prime }$ $\varepsilon_{c}^{\prime }=\frac{1.7f_{c}^{\prime }}{E_{c}}$ Check the neutral axis depth $c=\frac{A_{s}f_{s}+t_{f}b\varepsilon_{fe}E_{F}}{\alpha_{1}f_{c}^{\prime }\beta_{1}b}$	$\varepsilon_{c}^{\prime }=\frac{1.7(27)}{24400}=0.0019$, $\beta_{1}=\frac{4(0.0019)-0.0021}{6(0.0019)-2(0.0021)}=0.77$ $\alpha_{1}=\frac{3(0.0019)(0.0021)-0.0021_{}^{2}}{3(0.77)(0.0019^{2})}=0.92$ At N2 section: $c_{N}=\frac{\left(355)(410)+(1)(1000)(0.0072)(65100\right)}{\left(0.92)(27)(0.77)(1000\right)}=32.3\;\mathrm{mm}$ (OK) At mid-span section: $c_{P}=\frac{\left(355)(410)+(1)(1000)(0.0073)(65100\right)}{\left(0.92)(27)(0.77)(1000\right)}=32.7\;\mathrm{mm}$ (OK) Assumption of *ε* is satisfied.
9. Compute flexural strength at N2 section provided by - Steel: $M_{n,Ns}=A_{s,N}f_{s,N}(d-\frac{\beta_{1}c_{N}}{2})$ - CFRP: $M_{n,Nf}=t_{f}b\varepsilon_{fe}E_{F}\left(d_{f}-\frac{\beta_{1}c_{N}}{2}\right)$	$M_{n,Ns}=\frac{\left(355)(410\right)}{10^{6}}\left(120-\frac{\left(0.77)(32.3\right)}{2}\right)=15.64\;\mathrm{kNm}$ $\begin{array}{l} M_{n,Nf}=\frac{\left(1)(1000)(0.0072)(65100\right)}{10^{6}}\left(150.6-\frac{\left(0.77)(32.3\right)}{2}\right)\\ \;\;\;\;=64.25\;\mathrm{kNm} \end{array}$
10. Compute flexural strength at mid-span section provided by - Steel: $M_{n,Ps}=A_{s,P}f_{s,P}\left(d-\frac{\beta_{1}c_{P}}{2}\right)$ - CFRP: $M_{n,Pf}=A_{f,P}f_{fe,P}\left(d_{f}-\frac{\beta_{1}c_{P}}{2}\right)$	$M_{n,Ps}=\frac{\left(355)(410\right)}{10^{6}}\left(120-\frac{\left(0.77)(32.7\right)}{2}\right)=15.64\;\mathrm{kNm}$ $\begin{array}{l} M_{n,Pf}=\frac{\left(1)(1000)(0.0073)(65100\right)}{10^{6}}\left(150.6-\frac{\left(0.77)(32.7\right)}{2}\right)\\ \;\;\;\;=65.31\;\mathrm{kNm} \end{array}$
11. The design flexural strength $\phi_{f}M_{n}=\phi_{f}\left(M_{ns}+\psi_{f}M_{nF}\right)$ $\phi_{f}=0.9;\;\psi_{f}=0.85$	At N2 section: $\phi_{f}M_{n,N}=0.9\left(15.64+(0.85)(64.25)\right)=63.23\;\mathrm{kNm}$ At mid-span section: $\phi_{f}M_{n,P}=0.9\left(15.64+(0.85)(65.31)\right)=64.04\;\mathrm{kNm}$
12. The design shear strength $\phi_{v}V_{n}=\phi_{v}\left(d\sqrt{f_{c}^{\prime }}\right)\frac{b}{6}$ $\phi_{v}=0.75$	$\phi_{v}V_{n}=\frac{0.75}{6}(120)\sqrt{27}=77.94\;\mathrm{kN}$
13. Failure mode	B-2, as shown in Figure 6
14. Ultimate failure load Equation (7) for B-2	$w_{f}=\frac{2(77.94)}{\left(1.15)(2.5\right)}=54.2\;\mathrm{kN}/m$

**Table 5:** The strengthened slab capacities for the design example.

Slabs	Failure Mode	$w_{f}(\mathrm{kN}/m)$		*t_FN_* (mm)	*t_FP_* (mm)
Existing slab	D-2	31.0	[100%]	-	-
Strengthened both sections	D-2	47.9	[155%]	0.12	0.12
Strengthened negative sections	D-3	47.1	[152%]	0.26	-

**Figure A1:**
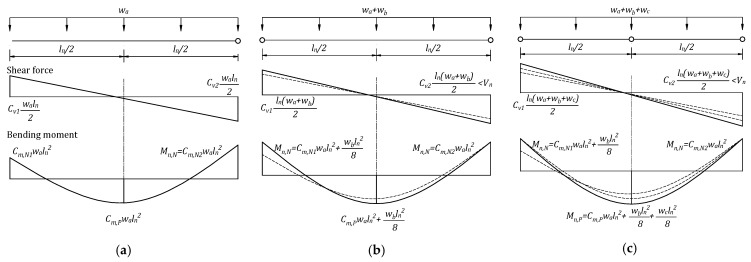
Failure mechanism for D-1 region. (**a**) Flexural failure at the N2 section first; next, (**b**) flexural failure at the N1 section; and finally, (**c**) flexural failure at the mid-span section.

**Figure A2:**
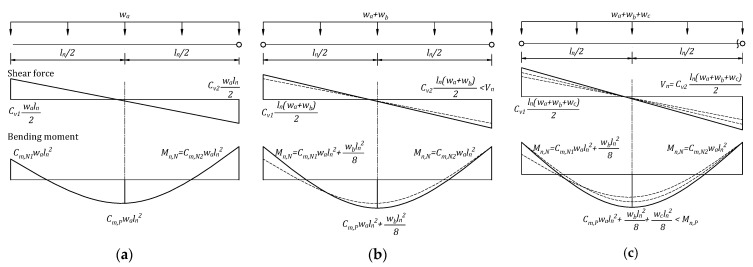
Failure mechanism for DB-1 region. (**a**) Flexural failure at the N2 section first; next, (**b**) flexural failure at the N1 section; and finally, (**c**) shear failure at the N2 section.

**Figure A3:**
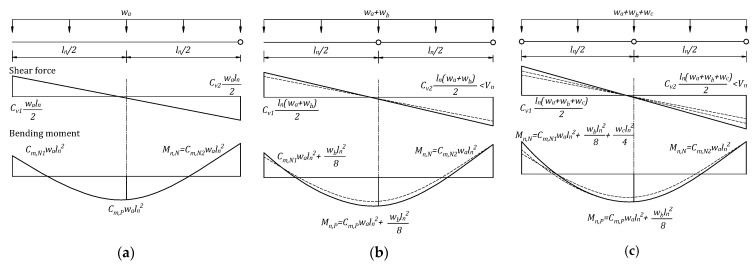
Failure mechanism for D-2 region. (**a**) Flexural failure at the N2 section first; next, (**b**) flexural failure at the mid-span section; and finally, (**c**) flexural failure at the N1 section.

**Figure A4:**
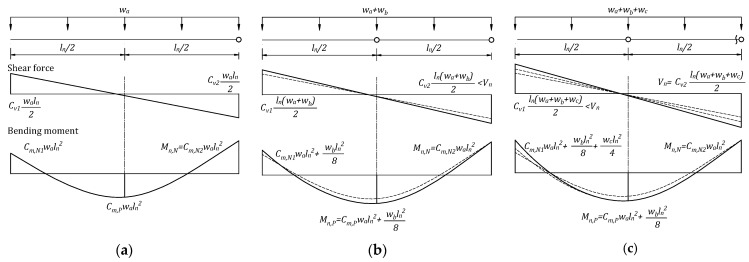
Failure mechanism for DB-2 region. (**a**) Flexural failure at the N2 section first; next, (**b**) flexural failure at the mid-span section; and finally, (**c**) shear failure at the N2 section.

**Figure A5:**
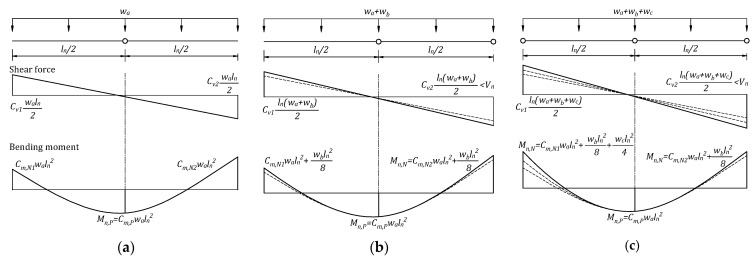
Failure mechanism for D-3 region. (**a**) Flexural failure at the mid-span section first; next, (**b**) flexural failure at the N2 section; and finally, (**c**) flexural failure at the N1 section.

**Figure A6:**
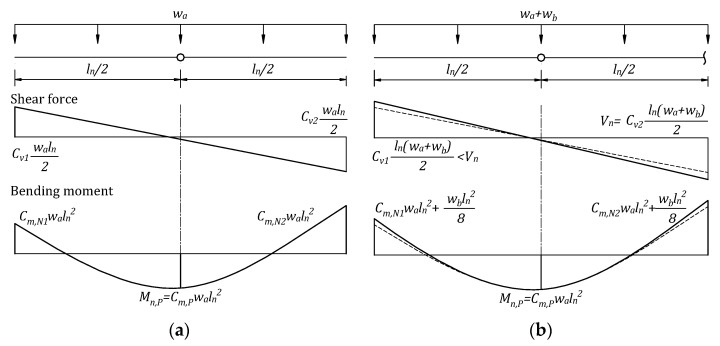
Failure mechanism for DB-3a region. (**a**) Flexural failure at the mid-span section first and then (**b**) shear failure at the N2 section.

**Figure A7:**
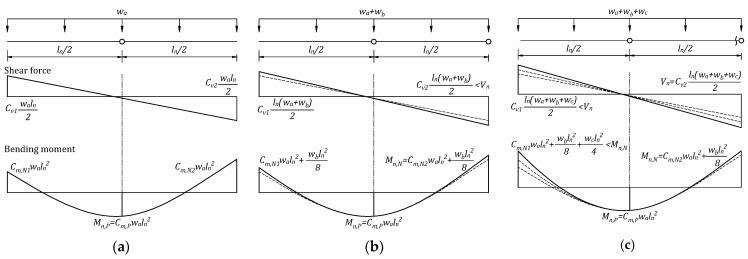
The failure mechanism for DB-3b region. (**a**) Flexural failure at the mid-span section first; next, (**b**) flexural failure at the N2 section; and finally, (**c**) shear failure at the N2 section.

**Figure A8:**
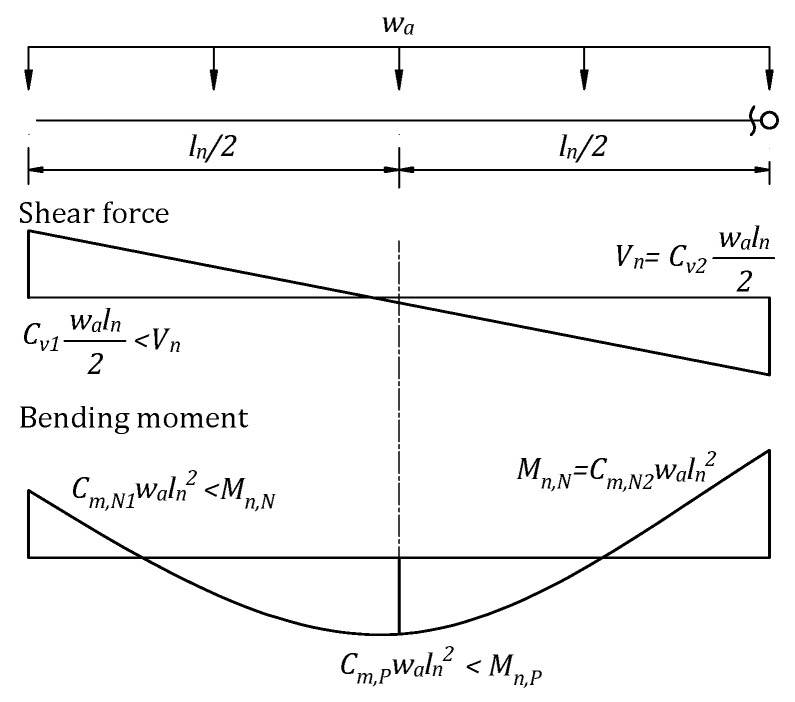
The failure mechanism for B-1 region; shear failure at N2 section after forming a plastic hinge at the N2 section.

**Figure A9:**
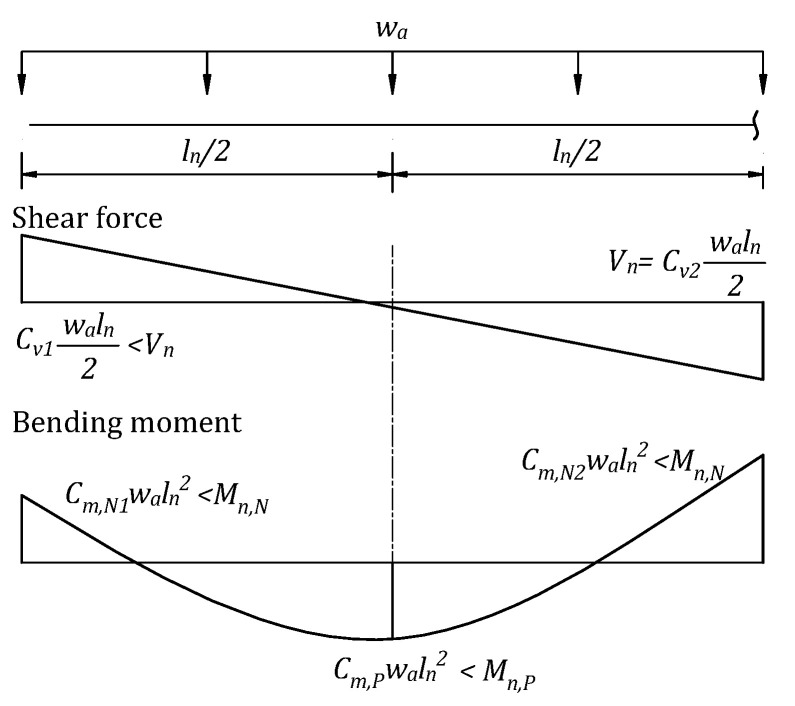
The failure mechanism for B-2 region; shear failure at N2 section without forming any plastic hinge.

## Data Availability

Not applicable.

## Appendix A

Details of the analysis for failure regions of Figure 3.

Region I: At Region I, where $M_{n,P}<2C_{m,P}V_{n}l_{n}/C_{v2}\;\mathrm{and}\;M_{n,N}<2C_{m,N1}V_{n}l_{n}/C_{v2}$, shown in Figure 3 includes a balanced flexural failure, the mid-span and the N1 sections fail simultaneously after the first plastic hinge forming at the N2 section as the applied load reaches its limit value. The sections at the mid-span and the N1 fail simultaneously when the condition in Equation (A1) is satisfied.

(A1) $$\frac{M_{n,N}}{M_{n,P}}=\frac{C_{m,N1}}{C_{m,P}}$$

When the left term in Equation (A1) is less than the right term, the first plastic hinge is formed at N2 section to warn of impending failure, followed by the second hinge at N1, and the ultimate slab failure can be observed once the third one is developed at the mid-span section, as shown in Figure A1. This failure is called “D-1”. In addition, the sections at the mid-span and the N2 fail simultaneously can occur when the condition in Equation (A2) is satisfied.

(A2) $$\frac{M_{n,N}}{M_{n,P}}=\frac{C_{m,N2}}{C_{m,P}}$$

When the left term in Equation (A2) is greater than the right term, a plastic hinge is formed at the mid-span section first, followed by the second hinge at the N2 section, and finally, the ultimate failure of the slab will occur upon the appearance of an additional plastic hinge at the N1 section as shown in Figure A5. This failure is called “D-3”. In the rest of the region I in Figure 3,

(A3) $$\frac{C_{m,N1}}{C_{m,P}}<\frac{M_{n,N}}{M_{n,P}}<\frac{C_{m,N2}}{C_{m,P}}$$

In case the conditions in Equation (A3) are satisfied, the formation of the first hinge at the N2 section is recognized, followed by the second hinge at the mid-span section, and the ultimate slab failure can be observed once the third hinge is formed at the N1 section, as shown in Figure A3. This failure is called “D-2”.

Regions II, III, and V: For Regions II, III, and V in Figure 3, either the flexural failure or the flexural−shear failure at sections of the RC slab can be recognized. In Region II, where $M_{n,P}>2C_{m,P}V_{n}l_{n}/C_{v2}\;\mathrm{and}\;M_{n,N}<2C_{m,N1}V_{n}l_{n}/C_{v2}$, the support sections fail and form the first plastic hinge at the N2 section. The value of the applied load ($w_{f}$) forming the first plastic hinge at the N2 section is calculated as follows,

(A4) $$w_{a}=\frac{M_{n,N}}{C_{m,N2}l_{n}^{2}}$$

After forming the first plastic hinge at the N2 section, it is subjected to *M_n,N_*. At the same time, the N1 section is subjected to the moment of $M_{N1}=C_{m,N1}w_{a}l_{n}^{2}.$ Considering the remaining flexural strength of the N1 section, $\left(M_{n,N}-M_{N1}\right)$, the value of additional distributed load to form a hinge at the N1 can be predicted.

(A5) $$w_{b}^{M}=\frac{8\left(M_{n,N}-M_{N1}\right)}{l_{n}^{2}}$$

Consequently, the N1 section, which holds the moment *M_n,N_*, cannot take any more load. At the same time, the mid-span section is subjected to the moment of $M_{P}=C_{m,P}w_{a}l_{n}^{2}+w_{b}l_{n}^{2}/8$, and the N2 section is subjected to the shear force of $V_{N2}=C_{v2}\left(w_{a}+w_{b}\right)l_{n}/2$. In case the additional moment at the mid-span section controls the ultimate failure load of the slab, as shown in Figure A1c, and considering the remaining flexural strength of the mid-span section, $\left(M_{n,P}-M_{P}\right)$, the additional distributed load capacity may be computed.

(A6) $$w_{c}^{M}=\frac{8\left(M_{n,P}-M_{P}\right)}{l_{n}^{2}}$$

The shear force at the N2 section, subjected to a uniformly distributed load, is always higher than that at the N1 section. Thus, the N2 section will fail in shear if the additional shear force at the support section controls the ultimate failure load of the slab as shown in Figure A2c, and the additional distributed load is calculated as

(A7) $$w_{c}^{V}=\frac{2\left(V_{n,N}-V_{N2}\right)}{l_{n}}$$

In this case, plastic hinges at the support sections will be formed before the slab fails in shear. The design limit could be established as

(A8) $$w_{c}^{M}=w_{c}^{V}$$

If $w_{c}^{M}$ in Equation (A6) is less than $w_{c}^{V}$, the failure mode will be D-1 described in Figure A1, while $w_{c}^{M}$ in Equation (A6) is greater than $w_{c}^{V}$, and two plastic hinges at the N1 and N2 sections can be developed before the slab fails in shear. This failure mode is called “DB-1” and is shown in Figure A2. By using M_P_ and V_N2_ in Equations (A6) and (A7) and using limit design in Equation (A8), one can find the design limit at Region II as follow,

(A9) $$M_{n,P}+M_{n,N}\left(\frac{C_{v2}/8+C_{m,N1}-C_{m,P}-C_{v2}C_{m,N1}}{C_{m,N2}}+C_{v2}-1\right)=\frac{1}{4}V_{n}l_{n}$$

For Region III, the two conditions $M_{n,P}<2C_{m,P}V_{n}l_{n}/C_{v2}\;\mathrm{and}\;2C_{m,N1}V_{n}l_{n}/C_{v2}<M_{n,N}<2C_{m,N2}V_{n}l_{n}/C_{v2}$ are applied. The mid-span section and the N2 section will fail by forming two plastic hinges, including a balanced flexural, where the sections of the mid-span and N2 fail simultaneously as the ultimate failure load (w_a_). This failure mode occurs when the condition in Equation (A2) is satisfied. When the left term appearing in Equation (A2) is less than the right term, the first plastic hinge will form at the N2 section as shown in Figure A3a at the amount of the applied load,

(A10) $$w_{a}=\frac{M_{n,N}}{C_{m,N2}l_{n}^{2}}$$

Hence, the N2 section, holding the moment *M_n,N_*, cannot take any more load. Simultaneously, the moment of the mid-span section is $M_{P}=C_{m,P}w_{a}l_{n}^{2}$. Considering the remaining flexural strength of the mid-span section, the additional distributed load capacity could be calculated, $\left(M_{n,P}-M_{P}\right),$ as

(A11) $$w_{b}^{M}=\frac{8\left(M_{n,P}-M_{P}\right)}{l_{n}^{2}}$$

After that, the mid-span section, holding the moment *M_n,P_*, cannot take any more load. Concurrently, the N1 section is subjected to the moment of $M_{N1}=C_{m,N1}w_{a}l_{n}^{2}+w_{b}l_{n}^{2}/8$, and the N2 section is subjected to the shear force of $V_{N2}=C_{v2}\left(w_{a}+w_{b}\right)l_{n}/2$ as shown in Figure A3b. In case the additional moment at the N1 section controls the ultimate failure load of the slab as shown in Figure A3c, the additional distributed load could be calculated by considering the remaining flexural moment of the N1 section, $\left(M_{n,N}-M_{N1}\right)$, as

(A12) $$w_{c}^{M}=\frac{4\left(M_{n,N}-M_{N1}\right)}{l_{n}^{2}}$$

The N2 section will fail in shear if the additional shear force at the support section controls the ultimate failure load of the slab as shown in Figure A4c. The additional distributed load is calculated as

(A13) $$w_{c}^{V}=\frac{2\left(V_{n,N}-V_{N2}\right)}{l_{n}}$$

In this case, plastic hinges at the N2 and the mid-span sections will be formed before the slab fails in shear. The design limit could be established as

(A14) $$w_{c}^{M}=w_{c}^{V}$$

If $w_{c}^{M}$ in Equation (A12) is less than $w_{c}^{V}$, the failure mode will be D-2 described in Figure A3, while $w_{c}^{M}$ in Equation (A12) is greater than $w_{c}^{V}$. Two plastic hinges at the N2 and the mid-span sections can be developed before the slab fails in shear. This failure mode is called “DB-2”, as shown in Figure A4. By using *M_N1_* and *V_N2_* in Equations (A12) and (A13) and using limit design in Equation (A14), one can find the design limit at Region III as follow,

(A15) $$M_{n,P}\left(2C_{v2}-1\right)+M_{n,N}\left(\frac{C_{v2}/4+C_{m,P}-C_{m,N1}-2C_{v2}C_{m,P}}{C_{m,N2}}+1\right)=\frac{1}{2}V_{n}l_{n}$$

When the left term appearing in Equation (A2) is greater than the right term, the mid-span section will fail first by forming a plastic hinge, followed by the second plastic hinge at the N2 section, and finally the third hinge at the N1 section. A similar failure mechanism, in this case, can be seen in Region V, where $M_{n,P}<2C_{m,P}V_{n}l_{n}/C_{v2}\;\mathrm{and}\;M_{n,N}>2C_{m,N2}V_{n}l_{n}/C_{v2}$; the mid-span section fails first and forms a plastic hinge as shown in Figure A5a at the amount of the applied load,

(A16) $$w_{a}=\frac{M_{n,P}}{C_{m,P}l_{n}^{2}}$$

Hence, the mid-span section, holding the moment *M_n,P_*, cannot take any more load. Concurrently, the N2 section is subjected to the moment of $M_{N2}=C_{m,N2}w_{a}l_{n}^{2}$ and the shear force of $V_{N2}=C_{v2}\left(w_{a}l_{n}/2\right).$ In Region III, the additional moment controls the ultimate failure load of the RC slab by forming a second plastic hinge at the N2 section, as shown in Figure A5b. In Region V, the ultimate failure load of the RC slab can be governed by either an additional moment or an additional shear force. If the additional moment at the N2 section controls the ultimate failure load of the RC slab, the additional distributed load is calculated by considering the remaining flexural moment of the N2 section, $\left(M_{n,N}-M_{N2}\right),$ as

(A17) $$w_{b}^{M}=\frac{8\left(M_{n,N}-M_{N2}\right)}{l_{n}^{2}}$$

If the ultimate failure load of the RC slab is governed by the additional shear force at the N2 section, as shown in Figure A6b, the additional distributed load is calculated.

(A18) $$w_{b}^{V}=\frac{2\left(V_{n,N}-V_{N2}\right)}{l_{n}}$$

In this case, only a plastic hinge at the mid-span section will be formed before the slab fails in shear at the N2 section. The design limit could be established as

(A19) $$w_{b}^{M}=w_{b}^{V}$$

If $w_{b}^{M}$ in Equation (A17) is less than $w_{b}^{V}$, the second hinge will be formed at the N2 section described in Figure A5b, while $w_{b}^{M}$ in Equation (A17) is greater than $w_{b}^{V}$, and a plastic hinge at the mid-span section can be recognized before the slab fails in shear. This failure is called “DB-3a” and is shown in Figure A6. Thus, considering the design limit in Equation (A19) with Equations (A17) and (A18), the design limit at Region V can be derived as follows,

(A20) $$M_{n,P}\left(\frac{C_{v2}/8-C_{m,N2}}{C_{m,P}}\right)+M_{n,N}=\frac{1}{4}V_{n}l_{n}$$

In case the second hinge forms at the N2 section after subjecting to the additional distributed load $w_{b}$, the N2 section cannot be subjected to any more load. Simultaneously, the N1 section is subjected to the moment of $M_{N1}=C_{m,N1}w_{a}l_{n}^{2}+w_{b}l_{n}^{2}/8$, and the N2 section is subjected to the shear force of $V_{N2}=C_{v2}\left(w_{a}+w_{b}\right)l_{n}/2$. In case the additional moment at the N1 section controls the ultimate failure load of the slab as shown in Figure A5c, the additional distributed load could be calculated by considering the remaining flexural moment of the N1 section, $\left(M_{n,N}-M_{N1}\right),$ as

(A21) $$w_{c}^{M}=\frac{4\left(M_{n,N}-M_{N1}\right)}{l_{n}^{2}}$$

The N2 section will fail in shear if the additional shear force at the support section controls the ultimate failure load of the slab as shown in Figure A7c, and the additional distributed load is calculated as

(A22) $$w_{c}^{V}=\frac{2\left(V_{n,N}-V_{N2}\right)}{l_{n}}$$

In this case, two plastic hinges at the mid-span section and the N2 section will be formed before the slab fails in shear. The design limit could be established as

(A23) $$w_{c}^{M}=w_{c}^{V}$$

If $w_{c}^{M}$ in Equation (A21) is less than $w_{c}^{V}$, the failure mode will be D-3, described in Figure A5, while $w_{c}^{M}$ in Equation (A21) is greater than $w_{c}^{V}$, and two plastic hinges at mid-span section and N2 section can be developed before the slab fails in shear. This failure is called “DB-3b” and is shown in Figure A7. Thus, considering the design limit in Equation (A23) with Equations (A21) and (A22), the design limit at Regions III and V can be derived as follows:

(A24) $$M_{n,P}\left(\frac{C_{v2}/4+C_{m,N2}-C_{m,N1}-2C_{v2}C_{m,N2}}{C_{m,P}}\right)+2C_{v2}M_{n,N}=\frac{1}{2}V_{n}l_{n}$$

Regions IV and VI: For Regions IV and VI in Figure 3, the slab will fail in shear, either forming a plastic hinge at the N2 section or without forming any plastic hinge. In Region IV, where $M_{n,P}>2C_{m,P}V_{n}l_{n}/C_{v2}$ and $2C_{m,N1}V_{n}l_{n}/C_{v2}<M_{n,N}<2C_{m,N2}V_{n}l_{n}/C_{v2},$ a plastic hinge at the N2 section can be recognized before the slab fails in shear at the same section as shown in Figure A8. This failure mode is called “B-1”. At Region VI, the two conditions $M_{n,P}>2C_{m,P}V_{n}l_{n}/C_{v2}\;\mathrm{and}\;M_{n,N}>2C_{m,N2}V_{n}l_{n}/C_{v2}$ are applied. The shear failure at the N2 section without forming any plastic hinge can be recognized, as shown in Figure A9. This failure mode is called “B-2”.
